# Evolution of the insect *Sox *genes

**DOI:** 10.1186/1471-2148-8-120

**Published:** 2008-04-26

**Authors:** Megan J Wilson, Peter K Dearden

**Affiliations:** 1 Laboratory for Evolution and Development, National Research Centre for Growth and Development, Department of Biochemistry, University of Otago, P.O. Box 56, Dunedin, New Zealand

## Abstract

**Background:**

The *Sox *gene family of transcriptional regulators have essential roles during development and have been extensively studied in vertebrates. The mouse, human and *fugu *genomes contain at least 20 *Sox *genes, which are subdivided into groups based on sequence similarity of the highly conserved HMG domain. In the well-studied insect *Drosophila melanogaster*, eight *Sox *genes have been identified and are involved in processes such as neurogenesis, dorsal-ventral patterning and segmentation.

**Results:**

We examined the available genome sequences of *Apis mellifera, Nasonia vitripennis, Tribolium castaneum*, *Anopheles gambiae *and identified *Sox *family members which were classified by phylogenetics using the HMG domains. Using *in situ *hybridisation we determined the expression patterns of eight honeybee *Sox *genes in honeybee embryo, adult brain and queen ovary. *AmSoxB *group genes were expressed in the nervous system, brain and Malphigian tubules. The restricted localization of *AmSox21b *and *AmSoxB1 *mRNAs within the oocyte, suggested a role in, or that they are regulated by, dorsal-ventral patterning. *AmSoxC, D *and *F *were expressed ubiquitously in late embryos and in the follicle cells of the queen ovary. Expression of *AmSoxF *and two *AmSoxE *genes was detected in the drone testis.

**Conclusion:**

Insect genomes contain between eight and nine *Sox *genes, with at least four members belonging to *Sox *group B and other *Sox *subgroups each being represented by a single *Sox *gene. Hymenopteran insects have an additional *SoxE *gene, which may have arisen by gene duplication. Expression analyses of honeybee *SoxB *genes implies that this group of genes may be able to rapidly evolve new functions and expression domains, while the combined expression pattern of all the *SoxB *genes is maintained.

## Background

The SOX gene family is a group of related transcription factors that play critical roles in embryonic development. This family was originally identified in mammals based on sequence similarity to SRY, the sex-determining region Y chromosome [1]. SOX proteins regulate gene expression by binding to DNA via a conserved DNA binding domain, the HMG (high mobility group) box (reviewed in [2]). Phylogenetic studies have determined that SOX family members segregate into ten groups (named A-J) on the basis of sequence similarities within the HMG box [3-5], with many groups containing multiple members from the same organism with related gene function. Human and mouse genomes each encode 20 *Sox *genes [3,6] and analysis of the genomes of many model organisms including chicken, *Drosophila*, Xenopus and Zebrafish reveal that the *Sox *gene family is conserved between animal phyla. Recently *Sox *genes have been identified in the genomes of the cnidarian *Nematostella vectensis*, ctenophores, and the sponge species *Reniera *indicating these are ancestral animal genes [7-9]. In vertebrates, SOX proteins have been shown to have essential roles in the formation of many body systems including the central nervous system, eye and heart development, bone cartilage, vasculature, sex determination and testis development [10-14]. Molecular and biochemical studies have shown that SOX proteins regulate cell fate and differentiation during development. Mutations in *Sox *genes have been shown to be the underlying cause of a number of human disorders and *Sox *genes are expressed during cancer progression [12,15-18].

In the arthropod model organism, *Drosophila melanogaster*, eight *Sox *genes have been identified and their expression patterns determined [19,20]. *Drosophila Sox *genes are expressed in the brain, developing eye, hindgut, nervous system and testes. Group B SOX proteins are present in the developing *Drosophila *central nervous system (CNS), and also in the CNS of vertebrates, implying that some *Sox *genes maintain a conserved role throughout evolution [19,21]. The phenotypes of *Drosophila Sox *gene mutants indicate that *Sox *genes are involved in dorsal-ventral patterning, segmentation and neurogenesis [22-28]. Collectively, these studies demonstrate that the SOX family is an evolutionally conserved group of proteins essential for development. In insects, however, only the *Sox *genes from *Drosophila *have been characterised.

Recent whole-genome sequencing projects for *Apis mellifera *(the honeybee), *Nasonia vitripennis *(a parasitic wasp), *Tribolium castaneum *(the red flour beetle) and *Anopheles gambiae *(the malaria mosquito) have been completed or are near completion [8,9,29-32] allowing the identification and classification of the complete complement of a gene family from several holometabolous insects. Here we identify *Sox *gene family members in the genomes of these insects and examine their relationship through phylogenetics. Additionally, we study the expression of the honeybee *Sox *genes by *in situ *hybridisation and RT-PCR.

An advanced social insect, the honeybee is fast becoming an important model organism for the study of behaviour, longevity, learning and memory, immunity, polyphenisms, evolution and development. Recently the honeybee genome has been sequenced [29] and analysis of developmental genes has revealed that some early acting developmental genes are absent [33]. Furthermore, the development of molecular techniques including *in situ *hybridisation and RNA interference (RNAi) [34-37] allow us to examine gene expression and the biological role of genes during honeybee embryogenesis and development, about which little is known despite the importance of the honeybee both scientifically and economically. Given the wide range of organ systems in which *Sox *genes are expressed, we aimed to identify and examine the expression of honeybee *Sox *genes. We identified nine *Sox *genes and used *in situ *hybridisation and RT-PCR to determine their expression patterns in the honeybee embryo, ovary and adult brain.

## Results

### Identification and relationships of insect *Sox *genes

**Figure 1:**
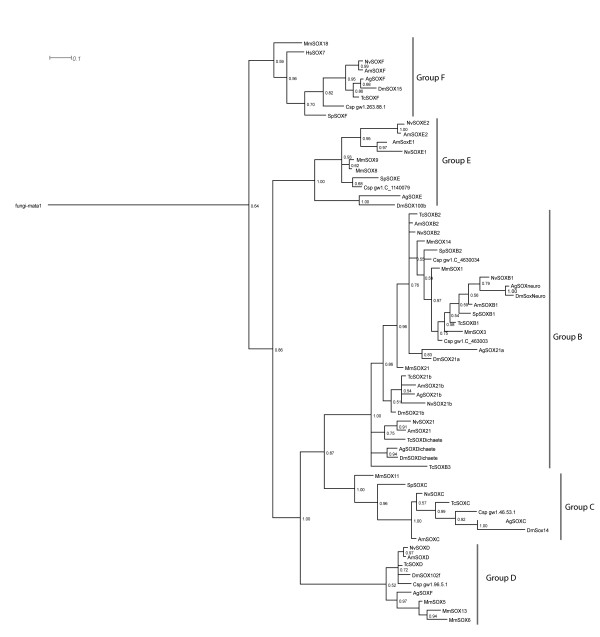
**Phylogeny of metazoan Sox proteins based on alignment of their HMG domains**. Phylogram drawn from Bayesian phylogenetic analysis of HMG domains. The tree was rooted with an established outgroup for SOX phylogenetics, Fu-MATA1 [3, 60]. The SOX proteins are subdivided into established subgroups (B-F). Abbreviations are fungi (Fu), *Drosophila melanogaster *(Dm), *Apis mellifera *(Am) and *Mus musculus *(Mm)*Nasonia vitripennis *(Nv), *Anopheles gambiae *(Ag) and *Tribolium castaneum *(Tc) *Capitella sp I *(Csp), *Strongylocentrotus purpuratus *(Sp).

BLAST searches [38] of the honeybee genome sequence with the SOX HMG box consensus sequence identified nine regions that encode homology to SOX proteins. We designated the genes encoding this homology *AmSox*. Each predicted *AmSox *gene was examined for the presence of a single HMG box and the sequence motif, RPMNAFMVW, conserved in all known SOX genes [3], to confirm that they were members of the SOX family of HMG transcription factors. The SOX family is subdivided into groups (A-J) based on phylogenetic comparisons [3]. Phylogenetic analysis of HMG domains from each predicted AmSOX protein placed them into groups B through F (Fig. 1). This allowed us to name *AmSox *genes based on their placement within each SOX groups. The honeybee genome has four group B *Sox *genes, two group E genes and one *Sox *gene for each of groups C, D and F. This data supports the notion that the major groups of *Sox *genes predate the separation of the lineages leading to Arthropods and vertebrates, as these groups exist in both lineages. Included in the tree as *Sox *gene sequences from a non-vertebrate deuterostome, the sea urchin *Strongylocentrus purpuratus*, and a lophotrochozoan, the annelid *Capitella sp I*. Examination of these genomes indicates that the different *Sox *gene classes are all present, in one copy, in these genomes. The only exception is that the sea urchin genome does not appear to contain a *SoxE *gene. As this class of *Sox *genes is found in diploblasts and all other metazoans, this must represent loss of this gene from the lineage leading to *Strongylocentrus*. SOX groups B-F are also found in the *Nematostella*, ctenophore and sponge genomes [7,8], indicating that the major groups of *Sox *genes predate the emergence of triploblastic animals.

**Figure 2:**
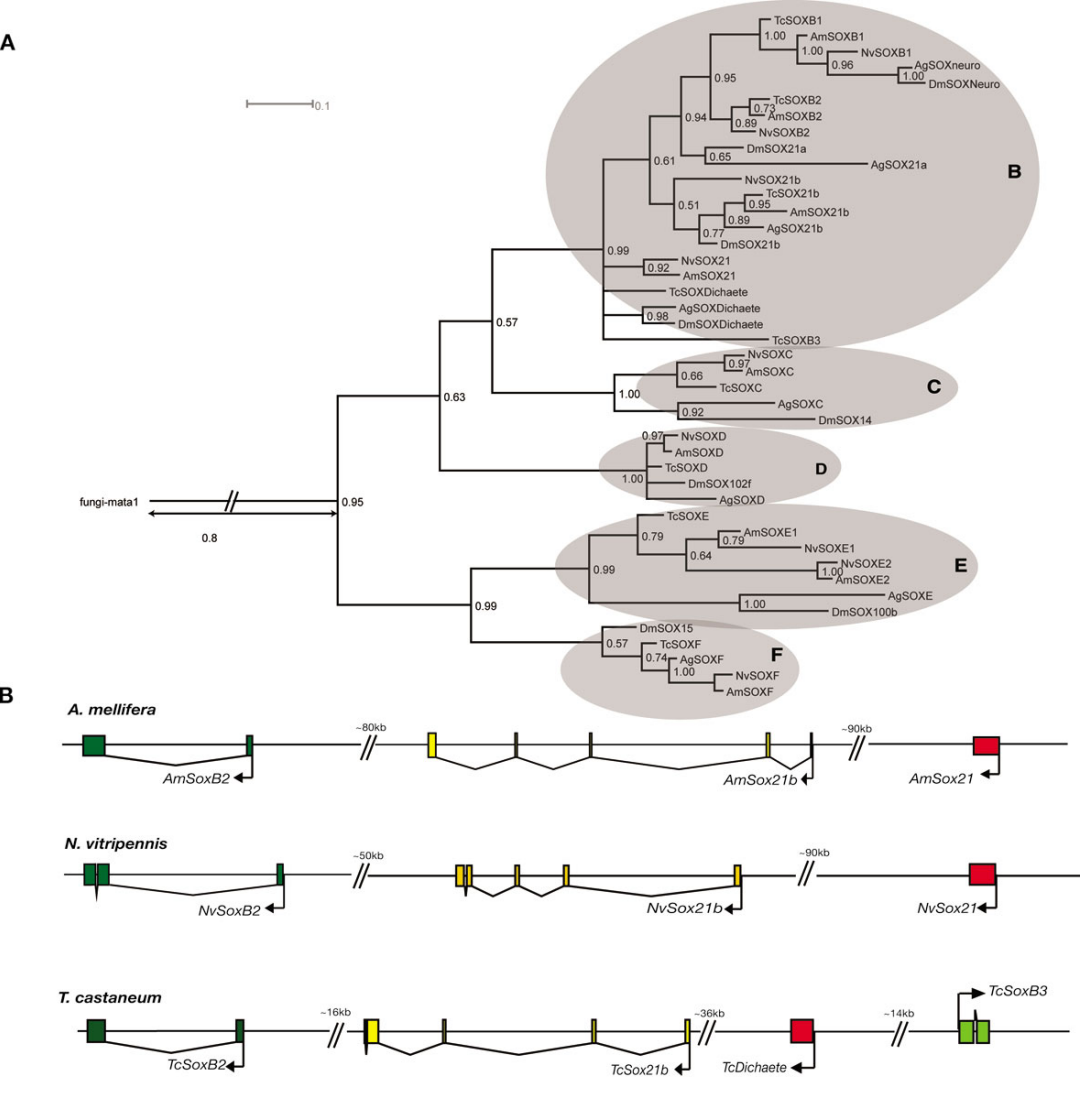
**Insect SOX HMG phylogeny**. A. A rooted Bayesian phylogeny for all identified SOX proteins encoded in *A. mellifera, D. melanogaster, A. gambiae, N. vitripennis *and *T. castaneum *genomes. SOX subgroups are indicated (B-F). B. An illustration of the genomic organisation of *Sox *group B genes in the honeybee, *Nasonia *and *Tribolium*. Exons are shown as coloured boxes with arrows indicating direction of transcription. Abbreviations are *Nasonia vitripennis *(Nv), *Apis mellifera *(Am), *Drosophila melanogaster *(Dm), *Anopheles gambiae *(Ag) and *Tribolium castaneum *(Tc).

To extend our analysis of insect *Sox *genes, we searched the publicly available insect genome projects for predicted SOX protein sequences. Eight *Sox *genes were identified in *Anopheles gambiae *and nine in both *Tribolium castaneum *and *Nasonia vitripennis *(Fig. 2A). In *Drosophila*, three *SoxB *genes are clustered within an 80 kb region, while *SoxB1 (SoxNeuro) *is located on a separate chromosome [20]. Similar arrangements are found in honeybees, *Anopheles *[20], *Nasonia *and *Tribolium *(Fig. 2B). *Tribolium *has five *SoxB *genes, four of which are clustered within a 90 kb region of the genome. In the honeybee and *Nasonia *there are large intergenic regions between each neighbouring *SoxB *gene and there are several predicted ORFs between *NvSox21b *and *NvSoxB2 *(Fig. 2B), suggesting they are unlikely to be co-regulated. In all cases, the *SoxB1/Neuro *orthologue is located in a different region of the genome. This confirms, as shown by [20], that there is an evolutionary conserved organisation of *SoxB *group genes between holometabolous insects, where at least three *SoxB *genes are located together in insect genomes (Fig. 2B).

In insects much of the diversity in *Sox *genes is found within the *SoxB *clade. Our phylogenetic analyses indicate that this clade is split into four groups (Fig. 2A), but that the details of the groupings are not well-resolved, due in part to the high sequence similarity of the HMG domains, and sequence divergence between SOX proteins outside of this domain. The SOX21/Dichaete clade is unresolved, but separate from the rest of the SOXB proteins. SOX21b proteins form a separate clade, as do the SOX21/Neuro orthologues. The SOXB2/21a clade is less well defined, with *Drosophila *SOX21a proteins being significantly different from SOXB2, perhaps indicating rapid evolution of these proteins in the lineage leading to Diptera.

**Figure 3:**
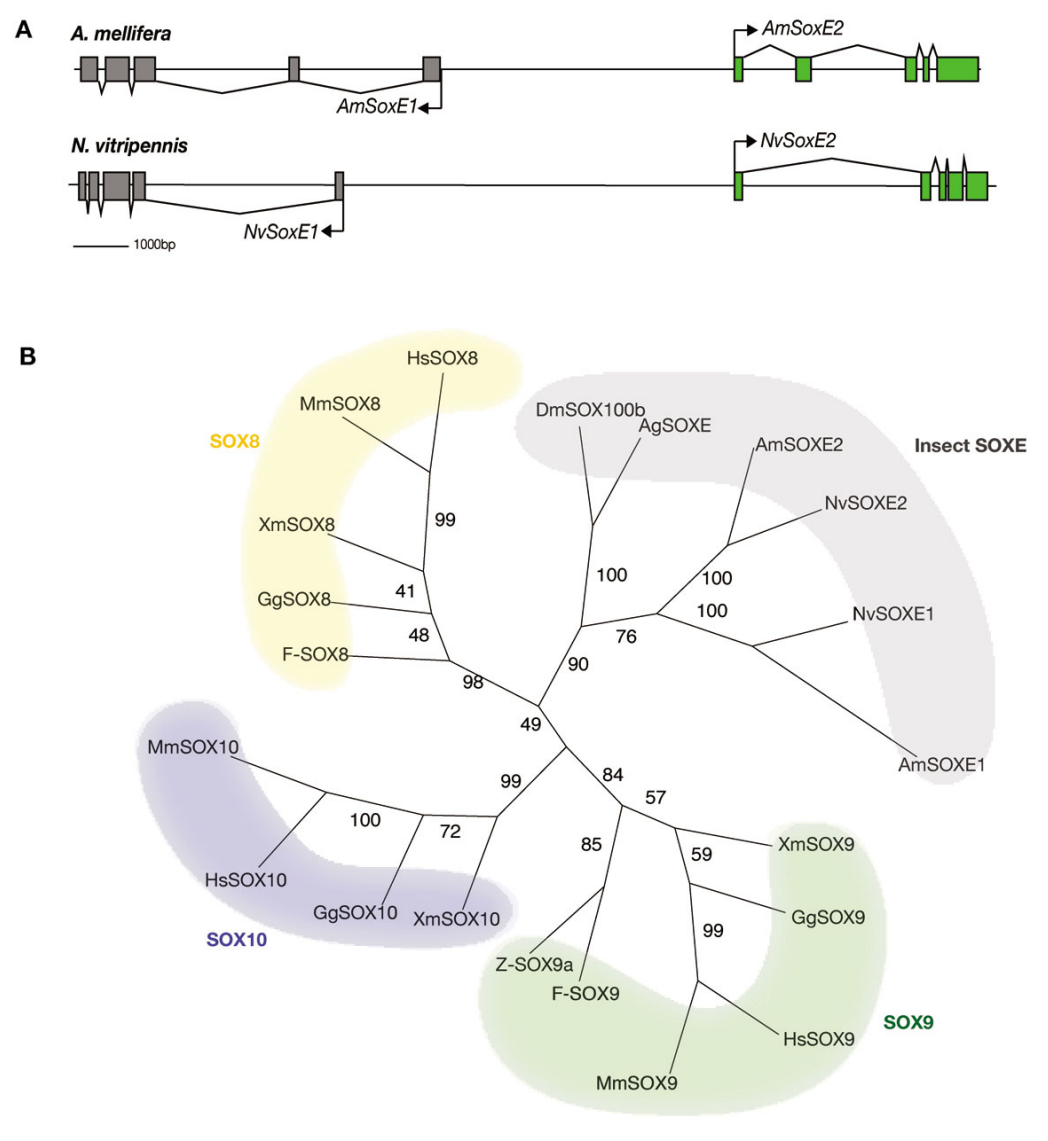
***SOXE *group gene duplication in hymenoptera**. A. Illustration of the *SoxE *gene genomic region from *A. mellifera *and *N. vitripennis *genomes. Both genomes encode two copies of *SoxE *group gene that share a common promoter region. B. Insect SOXE group proteins form a separate clade to the vertebrate SOXE proteins, that are split into three separate groupings, SOX8, SOX9 and SOX10. Insect SOXE proteins are most closely related to vertebrate SOX8 proteins. The unrooted tree was constructed using Phylip, bootstrap values are shown at internal branches.

Phylogenetic analyses revealed that honeybee and *Nasonia *both have one additional *Sox *group E gene compared to *Drosophila*, which has only one, *DmSox100b *(Fig. 2A). This additional SOXE protein seems likely to have arisen by gene duplication as both pairs of genes share a similar exon structure and appear to share a common promoter region (Fig 3A). This duplication must have occurred in an ancestor of hymenopteran insects before the split of *Nasonia *and *Apis*. Sequence analyses using full-length protein sequences revealed that invertebrate SOXE proteins form a clade separate to vertebrate SOXE proteins, and they are most closely related to the vertebrate SOXE protein, SOX8 (Fig 3B).

The phylogenetic analysis demonstrates the evolutionary stability of the *Sox *gene complement in insect evolution. The major amount of diversification in sequence appears in the *SoxB *group and in the duplication of the *SoxE *genes, that is seen only in hymenoptera. Given the stability in sequence we examined the expression of these genes in honeybees to determine if sequence stability is matched with constancy of predicted function.

### The expression patterns of *SoxB *group genes in the honeybee

Phylogenetic analysis reveals that the honeybee genome contains four group B *Sox *genes. These were also identified by McKimmie *et al*., [20], who investigated the genomic organisation of group *SoxB *genes in insects. We examined the expression patterns of these *Sox *genes in the queen ovariole, honeybee worker embryos and adult brains.

**Figure 4:**
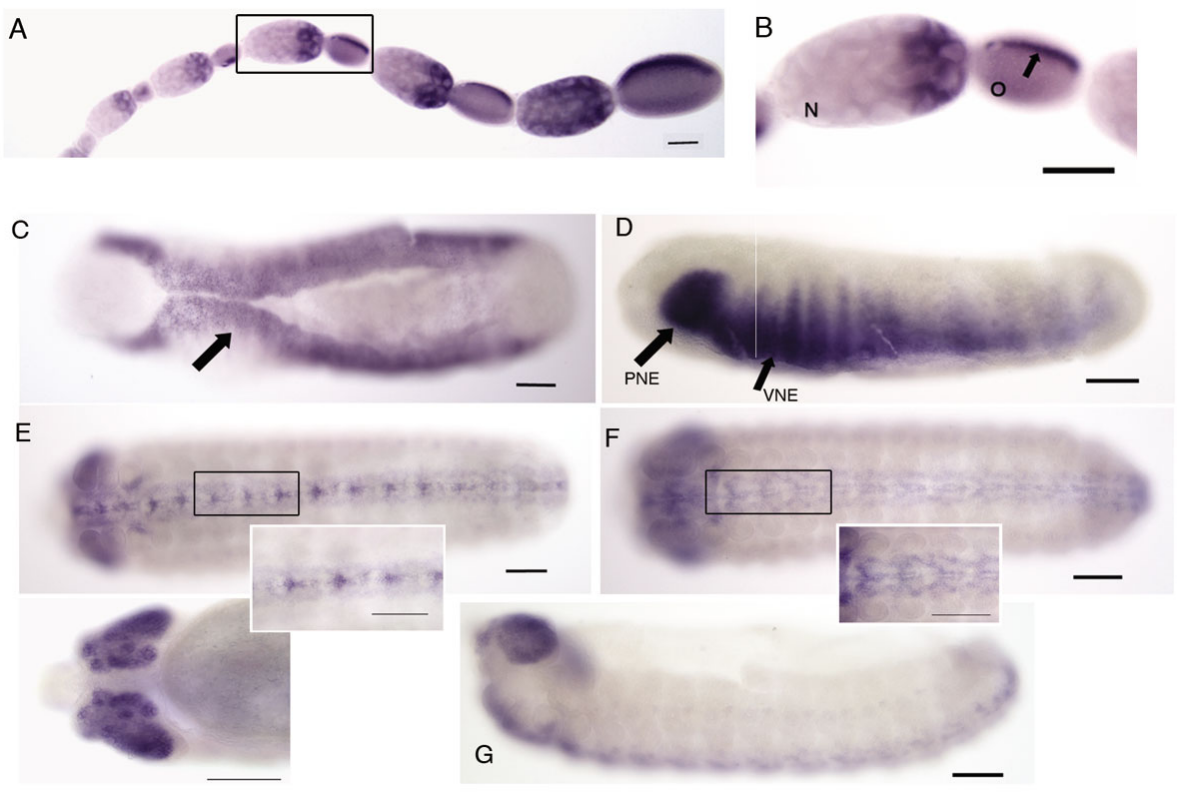
***AmSoxB1 *mRNA is localised in honeybee ooctyes and is expressed in the ventral neuroectoderm during honeybee embryogenesis**. A. Ovariole from honeybee queen was stained for *AmSoxB1 *mRNA. B. A higher magnification image of *AmSoxB1 *expression in the queen oocyte. *AmSoxB1 *mRNA is detected in the posterior nurse (N) cells and in the neighbouring oocyte, where the RNA becomes localized to the dorsal side of the oocyte (O; arrow). C. Ventral view of stage 6 embryo showing staining for *AmSoxB1 *along the gastrulation folds in the ventral neuroectoderm (VNE). D. Side view of later stage 6 embryo, *AmSoxB1 *is strongly expressed in the procephalic neuroectoderm. E. *AmSoxB1 *is expressed in the neuroblasts along the ventral midline at stage. *AmSoxB1 *expression in the cephalic brain lobes. F. In later stage embryos, neuronal expression of *AmSoxB1 *continues along ventral midline in neurons that radiate from the ventral nerve cord. Side view of the same embryo (G). Scale bar is 100 μm.

*AmSoxB1 *is strongly expressed by the nurse cells closest to the oocyte in the queen ovariole. In the oocyte, *AmSoxB1 *mRNA becomes localised to the dorsal surface (Fig. 4A and 4B). This expression pattern continues throughout oogenesis and *AmSoxB1 *mRNA is also detected on the dorsal surface of early (newly laid) embryos (data not shown).

During embryo development, *AmSoxB1 *is expressed along ventral gastrulation folds of stage 6 embryos (Fig. 4C), and in the procephalic neurogenic region. After gastrulation, *AmSoxB1 *expression continues in neuroblasts that arise from neuroectoderm along the ventral midline (Fig. 4E). At later stages these *AmSoxB1*-positive cells migrate to lateral positions along the ventral axis to differentiate and take up positions within the CNS. Strong expression of *AmSoxB1 *is also found in neurons of embryonic brain cephalic lobes. This expression continues in the brain of the adult worker honeybee, where *AmSoxB1 *continues to be expressed in Kenyon cells in each calyx of the mushroom bodies (data not shown), the key region of the honeybee brain required for sensory processing and memory formation.

**Figure 5:**
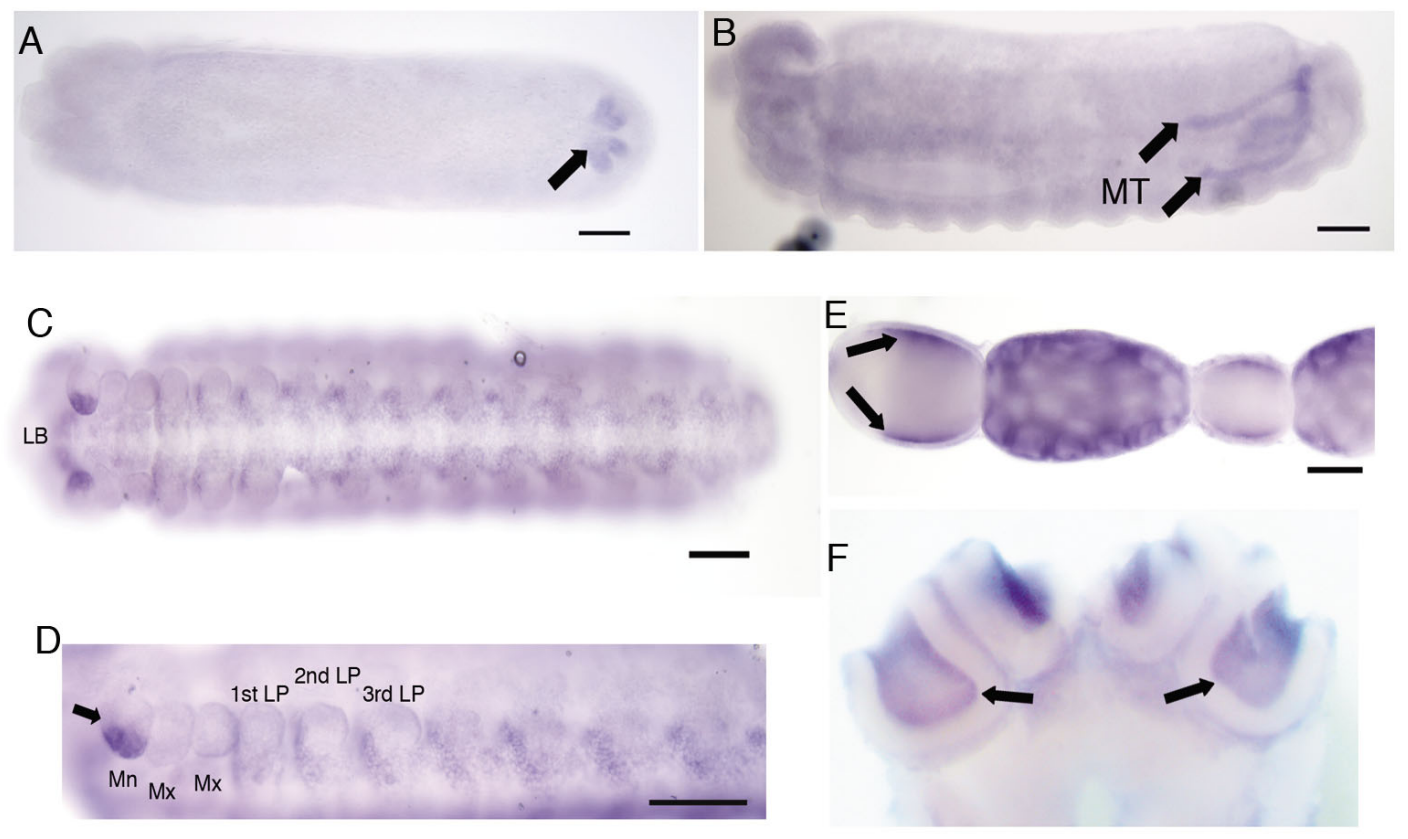
**AmSOXB2 and AmSOX21b expression in the honeybee**. *AmSoxB2 *mRNA is expressed during Malpighian tube development. A. Late stage 8, *AmSoxB2 *mRNA is detected at the posterior of the embryo (arrowed). B. In later stages, *AmSoxB2 *is present throughout the Malpighian tubules (MT). C. Stage 9 embryo showing *AmSox21b *staining in paired clusters of neurons that run adjacent to the ventral midline. B. Higher magnification (20x) of *AmSox21b *staining in mandible and CNS. C. *AmSox21b *mRNA is localized in the oocyte to the dorsal and ventral sides of the egg (arrowed). D. Expression of *AmSox21b *in the worker adult brain. *AmSox21 *mRNA is detected in the Kenyon cells of each calcyx of both mushroom bodies. Abbreviations; Mandible (Mn), Maxillary (Mx), leg pair (LP), labrum (LB). Scale bar = 100 μm.

*AmSoxB2 *expression is first detected in a group of cells at stage 8 in posterior region of the embryo, where the Malpighian tubules begin to form (Fig. 5A). This expression pattern is maintained in these tubules at later stages (Fig. 5B). AmSOXB2 is likely to have a role in the development of Malpighian tubules in the honeybee, which are essential for the removal of waste products and osmoregulation.

*AmSox21b *mRNA was detected in late embryos, in the CNS in paired/segmented ganglia on either side of the ventral nerve cord. Expression was also detected in the embryonic brain, intercalary head region and mandibles (Fig. 5C and 5D) and in the mushroom bodies of the adult worker brain (Fig. 5F). Strong *AmSox21b *expression is detected at the ventral tip of the developing mandible, implying that it may play a role in dorsal-ventral patterning of this appendage. In queen ovarioles, *AmSox21b *is strongly expressed by the nurse cells and its mRNA present in the oocyte, localized to both dorsal and ventral surfaces of the egg (Fig. 5E).

During late honeybee embryogenesis, the expression patterns of *AmSoxB1 *and *AmSox21b *group B genes do not overlap in the CNS (Figs 4F and 5C). These genes appear to be expressed in different neuronal cells along the ventral midline, implying that they play separate roles in the developing ÇNS. In the embryonic and adult brain, however, *AmSoxB1 *and *AmSox21b *are both expressed by the Kenyon cells of the mushroom bodies.

No expression was detected for *AmSox21 *by *in situ *hybridisation in honeybee embryos, queen ovaries or adult worker brains. *AmSox21 *is encoded by a single exon, making RT-PCR analysis of expression challenging, as RT-PCR analysis detects amplification from embryo cDNA but this is also seen in the control reaction in the absence of reverse transcriptase, indicating this band is most likely to be the result of genomic DNA contamination (Fig. 7). While previous studies [20] have identified *AmSox21 *in a brain EST library, an overlapping probe used in this study did not detect any expression. As *AmSox21 *expression is undetectable by RT-PCR and *in situ *hybridisation under our experimental conditions, we suggest that *AmSox21 *could be an inactive pseudogene. This implies the previously identified EST may be the result of genomic contamination.

**Figure 6:**
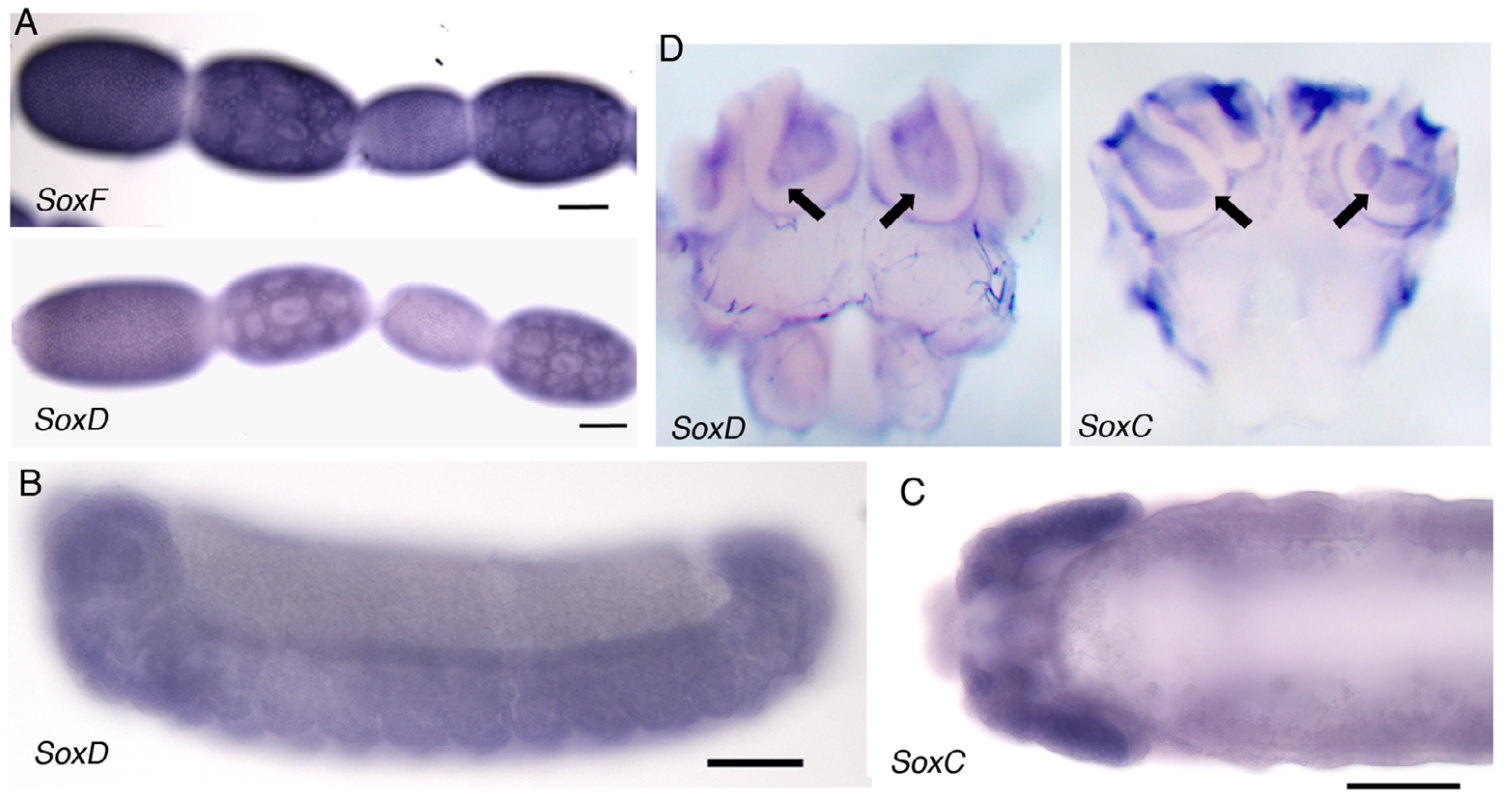
**Expression of *AmSoxC*, *AmSoxD *and *AmSoxF***. A. *AmSoxF *and *AmSoxD *expression was detected in the follicle cells surrounding each oocyte and all nurse cells in the queen ovariole. B. *AmSoxD *expression was unbiquous throughout late stage embryos. C. *AmSoxC *expression was stronger in the cephalic lobes of the honeybee embryo brain. D. *AmSoxC *and *AmSoxD *expression in the adult worker brain. Both are detected in the Kenyon cells of the mushroom bodies.

**Figure 7:**
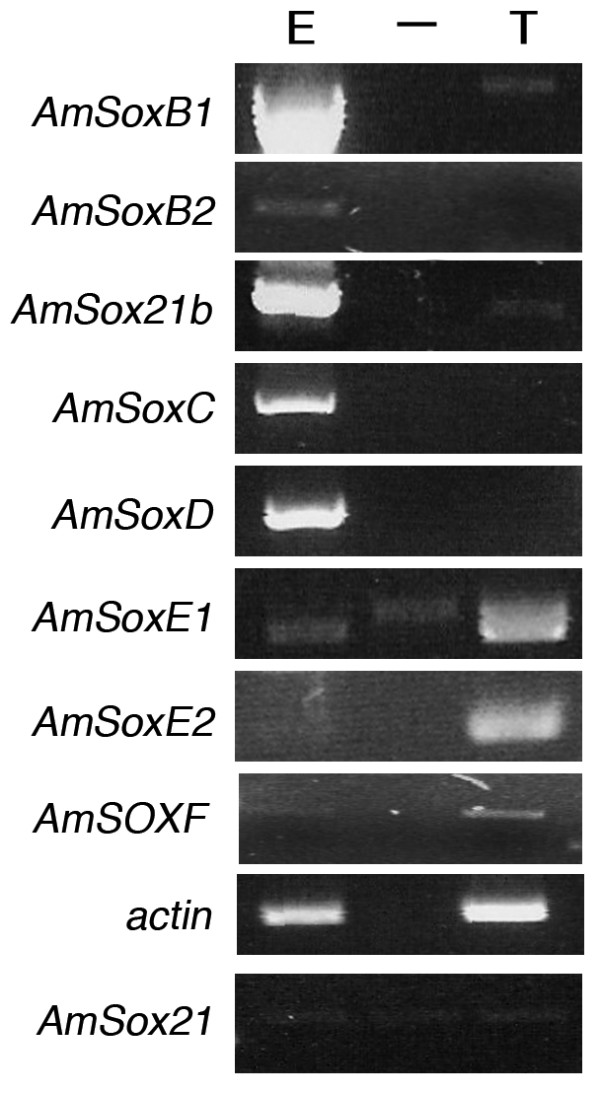
***AmSoxE1*, *AmSoxE2*, *AmSoxF *and *AmSox21b *are expressed in the testis of Drone honeybees**. Gene-specific primers were used to detect the presence of *AmSox *genes in total RNA isolated from whole honeybee worker embryos and Drone testis. A negative control reaction (no reverse transcriptase added to the cDNA synthesis) was performed for each set of oligonucleotide pairs to detect contamination from genomic DNA. Abbreviations: Embryo (E), testis (T) and negative control (—).

### Expression of *SoxF, D *and *C *group orthologues in the honeybee

*AmSoxC*, *AmSoxD *and *AmSoxF *were all expressed by nurse cells of the queen ovariole and the follicle cells that surround the oocyte (Fig 6A). All three were also expressed ubiquitously throughout late stage embryos (Fig. 6B), although *AmSoxC *expression was slightly higher in the embryonic brain (Fig. 6C). *AmSoxC *and *AmSoxD *were also expressed by the Kenyon cells in the calyces of the mushroom bodies (MB) (Fig. 6D),.

### *SoxE *group honeybee orthologues are upregulated in the drone testis

As SOX proteins play key roles in gonad differentiation, we used RT-PCR to determine if the honeybee *Sox *genes were also expressed in the testis of the drone (Fig. 7). RNA was isolated from the testis of drone pupa, as the adult drone testis degenerates shortly after emergence [39]. Strong expression of *AmSoxE1*, *AmSoxE2 *and *AmSoxF *was detected in testis and weak expression of *AmSox21b *(Fig. 7). *AmSoxF *was also expressed in queen ovaries (Fig. 6A) but only *AmSoxE *group gene expression appears to be testis-specific. No expression was detected in queen ovaries and only weak ubiquitous expression was found in late stage worker embryos.

## Discussion

We identified nine *Sox *genes in the honeybee genome, eight in *Tribolium*, seven in *Nasonia *and eight in *Anopheles*. Vertebrate genomes contain a much larger number of *Sox *genes; humans and mice have 20 *Sox *genes and fugu has 24 [6,40], with multiple *Sox *genes represented in each grouping, exhibiting overlapping expression patterns and functions. By contrast invertebrate deuterostome, ecdysozoan and lophotrochozoan genomes contain fewer *Sox *genes (8–9) and, apart from group B, only a single *Sox *gene represents most *Sox *groups. This is consistent with the hypothesis that the ancestral vertebrate genome underwent genome duplication(s) [41]. Non- bilaterian metazoa contain considerably more *Sox *genes [7,8] indicating *Sox *gene loss has been important in the evolution of the bilateria. While the HMG domain sequences of *Sox *group genes suggests that these genes are conserved, their expression when compared between *Drosophila *and honeybee indicates that these genes are evolving novel expression patterns and thus functions. *Sox *gene expression in *Drosophila*, *Apis *and vertebrates is summarised in Table 1.

**Table 1:** Summary of honeybee *Sox *group expression analysis.

Group	Honeybee expression summary	*Drosophila *expression summary	Vertebrate expression summary
**B**	*Adult brain*: MB* Embryo: neuroectoderm, CNS (ventral midline), brain, malpighian tubules, mandibles, intercalary. *Queen ovary*: oocyte and nurse cells.	*Embryo*: neuroectoderm, CNS, brain, hindgut, segments *Ovary*: oocyte and nurse cells	*Embryo*: CNS, lens, brain, stem cells, pituitary.
**C**	*Embryo: *ubiquitous expression *Adult brain*: MB *Queen ovary*: nurse and follicle cells of the ovary	*Embryo*: ubiquitous	*Embryo*: many tissues including CNS, heart, lung
**D**	*Embryo*: ubiquitous *Adult brain*: MB *Queen ovary*: nurse and follicle cells	*Embryo*: brain	*Embryo*: many tissues including chrondrocytes, spermatogenesis, CNS, brain, thymus, ovary
**E**	*Adult testis Embryo*: no expression detected	*Embryo*: alimentary canal, gonadal mesoderm	*Embryo*: CNS, brain, limbs, heart, testes, chondrocytes, kidney, neural crest
**F**	*Embryo: *ubiquitous *Queen ovary*: nurse and follicle cells	*Embryo*: peripheral nervous system	*Embryo*: endoderm, blood vessel and hair follicles.

* abbreviations: mushroom bodies (MB), central nervous system (CNS)

### Expression and evolution of *SoxB *genes

The general features of group B gene expression are conserved for the honeybee, as their expression patterns suggest roles in neurogenesis and dorsal-ventral patterning. However, orthology based on phylogenetic evidence does not predict the expression pattern of an individual gene. Despite conservation in genomic organisation and sequence in insects [20], expression of the individual *SoxB *genes has changed considerably through the evolution of insects.

None of the *AmSox *B group genes show identical expression patterns to any of their orthologous *DmSox *B genes. For example, the *AmSox21b *expression pattern in the CNS is different to that of *DmSox21b*, which is expressed in abdominal epidermal stripes. *AmSoxB1 *expression pattern overlaps with both *DmSoxB1 *and *DmDichaete *(*DmSoxB2.1*) expression patterns. No expression was detected for *AmSox21*, which had been suggested to be a orthologue of *DmDichaete *by McKimmie et al. [20] based on phylogenetics and genome position, and is *Dichaete's *nearest neighbour in our phylogenetic analysis.

Recently, in *Drosophila*, examination of a *DmDichaete *(*DmSoxB2.1*) loss of function mutant found that Dichaete influenced dorsal-ventral patterning [23]. Mutant eggs had defects in Gurken-dependent formation of dorsal appendages and differentiation of dorsal/anterior follicle cells. Additionally, in zebrafish, both knock-down and ectopic expression of the SOX protein SOX21a indicates that it acts in dorsal-ventral patterning [42]. In the honeybee, two *Sox *genes appear to have a role in, or a regulated by, dorsal-ventral patterning. *AmSoxB1 *mRNA is localized to the dorsal surface of the oocyte and *AmSox21b *mRNA is localized to both the dorsal and ventral surface of the oocyte. As mRNA localization plays a critical part in axis specification in other insects [43,44], it is likely that these *AmSox *genes have roles in dorsal-ventral patterning in the oocyte and they may have overlapping functions. It is currently unknown how axes are specified in the honeybee oocyte and early embryo, as the honeybee genome is missing several key genes essential for axis organisation in *Drosophila *[33]. While these expression patterns suggest a conserved role for SOX group B proteins in dorsal-ventral patterning, the actual *SoxB *genes involved are not orthologous. The direct orthologue of *DmDichaete*, according to our phylogenetic analysis (and that of [20]) is *AmSox21*, which has no expression in the oocyte, while the honeybee orthologues of zebrafish *Sox21a *are *AmSoxB2 *and *AmSox21a*.

We have also found a novel expression pattern for a group B SOX protein, AmSOXB2, in the formation of the Malpighian tubules. The *Drosophila *group E SOX protein (*DmSox100b*) is also expressed in Malpighian tubules, while SOX proteins in mammals are expressed in analogous tissues, the foetal kidneys [45]. AmSOXB2 sequence is highly divergent outside of the HMG box[20] perhaps reflecting different selective pressure on its sequence due its co-option into a possible role in Malphigian tubule formation.

### Other Sox genes

*AmSoxC*, *AmSoxD *and *AmSoxF *were expressed throughout late stage embryos. The *Drosophila *orthologue for *SoxC *is also ubiquitously expressed [19,46], although *SoxD *and *SoxF *orthologues show specific nervous system expression. Vertebrate *SoxD *orthologues are expressed broadly in embryonic tissues [47] and more specifically in bone and pancreas.

There is little conservation in expression of SOXF group members between species. Vertebrate *SoxF *family members are involved in a range of activities including endoderm specification, blood and hair follicle development. *DmSoxF *is found in the peripheral nervous system [19] whereas *C. elegans *does not have a *SoxF *group gene [3].

*Drosophila DmSox100b *is expressed in gonadal mesoderm and its expression becomes male-specific after stage 15 but it is also expressed in other tissues including the alimentary canal, intestinal cells and Malpighian tubules [45,48]. Upregulation of AmSOXE proteins solely in the drone testis implies that they may play a specific role in honeybee testis differentiation. Group F SOX proteins, a group closely related to SOXE proteins (Fig. 1), are also expressed in both testis and ovaries in other species including the eel [49] and human (*sox17*; [50]), indicating that SOXF proteins play a conserved evolutionary role in both male and female gonads.

Sequence analyses revealed the honeybee and *Nasonia *genomes encode two *SoxE *group members where-as there is only one in *Drosophila *(*DmSox100b*) and none in *C. elegans *[3]. Expression of both *AmSoxE *genes was upregulated in the testis of honeybee drones, suggesting they play a role in testicular development. SOXE group proteins are expressed during testis determination in many species [48,51-53]. Sequences outside of the HMG domains of SOXE1 and SOXE2 show little similarity. These sequence changes may have been necessary for interactions with other testis-related factors. Non-HMG domain sequences can play a role in protein partner selection between different SOX groups but SOX proteins within the same subgroup often interact with the same protein partners despite having sequences that are different outside of the HMG domain [54].

## Conclusion

We identified and classified *Sox *genes in the genomes of *Apis mellifera*, *Nasonia *and *Tribolium *and examined the expression patterns of eight honeybee *Sox *genes *by in situ *hybridisation. The expression patterns of honeybee *Sox *genes confirm that members of this family are likely to play an essential role in embryogenesis and neural specification. Further studies are required including knock-down of gene expression to confirm the predicted roles of *Sox *genes in the honeybee.

## Methods

### Phylogenetics

SOX homologues were identified in insect genome sequences using tBlastN searches [38]. Each putative AmSOX protein was analysed for the presence of a sequence motif RPMNAFMVW located within the HMG box which is conserved for all SOX sequences [3], confirming that those genes identified were members of the SOX group of HMG domain transcription factors. Multiple alignments of honeybee SOX HMG sequences with SOX domains from other species were carried out in ClustalX (see Additional files 1, 2 and 3). For Figure 1 the multiple alignment was analysed using MrBAYES 3.1.2.[55] under the WAG model[56] with default priors. The WAG model was chosen as the most appropriate model of amino-acid substitution after preliminary analysis using MrBAYES with mixed models. The Monte Carlo Markov Chain search was run with four chains over 1500000 generations with trees sampled every 1000 generations. The first 375000 trees were discarded as 'burn-in'. The trees in figures 2 and 3 were constructed using the PHYLIP[57] package of programs from alignments bootstrapped using SEQBOOT. Maximum Likelihood trees were estimated using PROTML and majority-rule consensus trees derived using CONSENSE. Dendrograms were displayed using TreeViewPPC [58] or Dendroscope [59].

Genome sequence information for insects and other species was retrieved from their genome project websites [[32,61-63] and [64]]. Exon/intron gene structure was predicted by either Genemachine [65] or was already predicted during the genome assembly using sets of reference sequences (including *Drosophila*) to help identify transcripts. Insect *Sox *genes were named based on their placement within each SOX groups (see Additional file 4 for Genebank accession numbers).

### Isolation of *AmSox *gene probes

Total RNA was extracted from Honeybee embryos or testis dissected from drone pupa using the RNeasy Mini Kit (Qiagen) and cDNA was produced using Superscript II reverse transcriptase (Invitrogen). *AmSox *gene fragments were amplified by RT-PCR from embryo cDNA using oligonucleotide primers corresponding to non-HMG box encoding regions from the coding sequence of each predicted *AmSox *gene. Oligonucleotide primers used were: *SoxC *– 5'AGAAGCTGAGGAAATCGGGT3' and 5'AATTCCATCTTCATCTTTCCGTC3'; *SoxB1 *– 5'GCTCAAGAAGGATAAATTCCCC3' and 5'AATCGCCGTGTGATGCTG3'; *soxB2 *– 5'TCACACGTTGATGAGCCAC3' and 5'GACGACGACAAATTCTCCTCTTC3'; *Sox21 *– 5'-TCCAGGATCGAAGACCACC3' and 5'CTAGAATATTACGGAGACTGGCC3'; *Sox21b *– 5'GAAGTATTCGATGGAAGCGG3' and 5'GATGACAGTGAGCGGTCGT3';*SoxE1 *– 5'CCAGAGCAACGTGACTTTCA3' and 5'CCACCTCGCACTCCTGAA3'; *SoxE2 *– 5'GAACGCGTTCATGGTCTG3' and 5'TCCTCGTGCACCGTGTAC3'; *SoxF *– 5'CTGAATTCAGGAAGACCAGTGG3' and 5'GACGGCTGTCTCTCGAAATT3'; *SoxD *– 5'GGAAGAAGATGCGCATATCC3' and 5'-TCAATCCTCGTCGTGGTG. *Actin *was amplified using 5'CTCTCTTTGATTGGGCTTCG3' and 5'TGACGAAGAAGTTGCTGCAC3' oligonucleotide primers as a positive control for RT-PCR. Amplified *AmSox *DNA fragments were then cloned into the pGEM-T Easy vector (Promega). The sequence and orientation of each cloned gene fragment was confirmed by DNA sequencing.

### *In situ *hybridization

Honeybee embryos were collected and fixed as described [34]. Brains were dissected from worker honeybees, fixed in 4% PFA overnight at 4°C and stored in methanol. Anti-sense or sense digoxigenin (DIG)-labeled RNA probes were produced by *in vitro *transcription from linearized DNA templates containing *AmSox *cDNA fragments. *In situ *hybridization on honeybee embryos, oocytes and worker brains were performed as described [34].

## Authors' contributions

MJW conceived and designed the study, performed sequence analysis and honeybee expression studies, and drafted the manuscript. PKD participated in the design of the study and sequence analysis and helped to draft the manuscript.

## Supplementary Material

Additional file 1 **Multiple alignment of honeybee SOX and vertebrate HMG domains**. Alignment of the HMG domains from honeybee SOX proteins and vertebrate SOX proteins created in ClustalX. Click here for file

Additional file 2 **Multiple alignment of insect SOX HMG box sequences**. Alignment of the HMG domains from predicted insect SOX proteins created in ClustalX Click here for file

Additional file 3 **Multiple alignment of SOXE full length protein sequences**. ClustalX alignment of full-length SOXE proteins from insects and vertebrates. Click here for file

Additional file 4 **Naming of identified insect *Sox *genes**. Renamed insect SOX genes (based on phylogenetics) and their protein Genbank accession numbers. Click here for file
