# Supplementary material for: Evolution of the insect Sox genes
Source: BMC Evol Biol. 2008 Apr 26;8:120. doi: 10.1186/1471-2148-8-120 (PMC2386450; doi:10.1186/1471-2148-8-120)
Supplement: Additional file 4 — Naming of identified insect Sox genes. Renamed insect SOX genes (based on phylogenetics) and their protein Genbank accession numbers. [file 1471-2148-8-120-S4.doc]

| Gene annotation name/contig | SOX name |
| --- | --- |
| *Apis mellifera* |  |
| XP_001122996.1 | AmSOXE2 |
| XP_0011229993.1 | AmSOXE1 |
| XP_001121910.1 | AmSOXB2 |
| XP_001121956.1 | AmSOX21 |
| XP_001122045.1 | AmSOXF |
| XP_391958.3 | AmSOXB1 |
| XP_001122631.1 | AmSOXC |
| XP_001121937.1 | AmSOX21b |
| XP_392414.3 | AmSOXD |
| *Nasonia vitripennis* |  |
| XP_00603143.1 | NvSOXD |
| XP_001604932.1 | NvSOXE2 |
| XP_00160413.1 | NvSOXE1 |
| XP_001603867.1 | NvSOXF |
| XP_001606916.1 | NvSOXC |
| XP_001606472.1 | NvSOXB2 |
| XP_00160557.1 | NvSOX21b |
| Unannotated/NW_001818015.1 | NvSOX21 |
| Unannotated/NW_001816793.1 | NvSOXB1 |
| *Anopheles gambiae* |  |
| AgaP_AGAP010923 | AgSOX21a |
| AgaP_AGAP010922 | AgSOX21b |
| AgaP_AGAP010919 | AgSOXdichaete |
| AgaP_AGAP000922 | AgSOXE |
| AgaP_AGAP012033 | AgSOXF |
| AgaP_AGAP005183 | AgSOXC |
| AgaP_AGAP009957 | AgSOXneuro |
| AgaP_AGAP000066 | AgSOXD |
| *Tribolium castaneum* |  |
| XP_971910.1 | TcSOXB2 |
| XP_974496.1 | TcSOXB1 |
| XP_97072.1 | TcSOXB3 |
| XP_970919.1 | TcSOXdichaete |
| XP_973116.1 | TcSOXC |
| XP_971967.1 | TcSOX21b |
| XP_974207.1 | TcSOXF |
| Unannotated/aajj01002010.1 | TcSOXE |
